# Efficacy and safety of first-line combination therapy versus monotherapy for vitreoretinal lymphoma: a systematic review and meta-analysis

**DOI:** 10.1186/s12886-023-03226-3

**Published:** 2023-11-22

**Authors:** Jing Gao, Xiaoyan Peng, Liang Wang

**Affiliations:** 1 Department of Hematology, Beijing Tongren Hospital, Capital Medical University, Beijing, 100730 China; 2 Department of Ophthalmology, Beijing Tongren Hospital, Capital Medical University, Beijing, 100730 China

**Keywords:** Vitreoretinal lymphoma, Combination therapy, Monotherapy, BTK inhibitors, Meta-analysis

## Abstract

**Background:**

Vitreoretinal lymphoma (VRL) is usually treated with a combination of intraocular methotrexate (ioMTX), high-dose intravenous methotrexate (HD-MTX), or local radiotherapy (RT) as the first options. The effectiveness and safety of monotherapy like bruton’s tyrosine kinase inhibitors (BTKi) for PVRL remain uncertain.

**Methods:**

A systematic review and meta-analysis of clinical trial data and conference abstracts in VRL patients treated with first-line combination therapy or monotherapy were conducted through a search of PubMed, Embase, and Scopus databases until December 2022. A total of 24 studies comprising 517 patients were included, and survival data were extracted from 279 patients due to inconsistent units across studies.

**Results:**

The combined treatment group used ioMTX + chemotherapy (in 4 studies), RT + chemotherapy (in 2 studies), ioMTX/HD-MTX based regimen (in 2 studies), ioMTX + RT + chemotherapy (in 2 studies), ioMTX + lenalidomide/BTKi (in 2 studies) and combination of multiple therapies (in 7 studies). The monotherapy group was mainly treated with oral monotherapies such as BTKi. The combination therapy had a higher overall response rate (ORR) and complete response rate (CRR) than monotherapy (ORR: 96% vs. 72%, CRR: 92% vs. 63%). Combination therapy also resulted in a longer median progression-free survival (28.8 months vs. 13 months, *p* = 0.012). However, the combination therapy group had more severe side effects (grade 3/4 toxicity) than the monotherapy group (45% vs. 8%).

**Conclusion:**

The study showed combination therapy had better OR and CR rates, longer survival, and more toxicity than monotherapy. While BTK inhibitors were well-tolerated, long-term effectiveness needs confirmation from prospective studies. In addition, given the small number of studies of monotherapy for VRL, more studies are needed to validate its effects.

**Trial registration:**

CRD42023400305.

**Supplementary Information:**

The online version contains supplementary material available at 10.1186/s12886-023-03226-3.

## Introduction

Vitreoretinal lymphoma (VRL), also known as intraocular lymphoma (IOL), is a rare variant of central nervous system lymphoma (CNSL). It is a extranodal, non-Hodgkin's lymphoma, typically of the B-cell type, which predominantly affects the vitreous and retina of the eye, while also potentially involving the optic nerve without any infiltration of the brain parenchyma. It is important to note that VRL is an exceptionally aggressive lymphoma subtype, often posing significant challenges for diagnosis and treatment.

VRL is a very rare disease, with only approximately 50 new cases reported annually in the United States, mostly affecting elderly patients. Additionally, women appear to be more susceptible than men [1]. Nonetheless, there exists a close association between VRL and CNSL, as some CNSL may ultimately develop an ocular manifestation, while most VRL-origin lymphomas may eventually progress to CNSL. Hence, despite its low prevalence, the severity of VRL should not be underestimated, making it crucial to find an appropriate treatment strategy that can minimize the risk of CNS recurrence while alleviating ocular symptoms.

The first-line treatment for VRL typically includes both local treatment, such as intravitreal injection of chemotherapy, ocular radiotherapy, and systemic therapy based on high-dose (HD) methotrexate (MTX). Nevertheless, the contribution of the combination of these two first-line treatments to improved outcomes remains controversial [2]. In addition to local and systemic treatments, there are currently several other therapeutic modalities that have gained widespread attention in research. Various single agents, including temozolomide, and targeted agents such as Bruton tyrosine kinase inhibitors (BTKi), are also emerging as potential treatment options for VRL [3, 4]. However, the specific advantages and disadvantages between the two first-line therapy modalities and monotherapy remain unclear. Thus, the objective of this study is to conduct a systematic review and meta-analysis comparing the efficacy and safety of first-line combination therapy versus monotherapy, in order to provide recommendations for future clinical management.

## Methods

### Search strategy and selection criteria

This systematic review and meta-analysis adhered to a previously published protocol registered on the PROSPERO registry (CRD42023400305) and followed the PRISMA (Preferred Reporting Items for Systematic Reviews and Meta-Analyses) guidelines [5]. A comprehensive search of the literature was conducted to identify articles published in PubMed, Embase, and Scopus up to December 2022. Furthermore, relevant data from conference abstracts were included in the analysis if available. The complete search algorithm is provided in Supplementary Table 1. The outcome measure of interest is the median progression-free survival, which is defined as the duration of time during which 50% of patients remain free of disease progression.

The inclusion criteria for this review consisted of the following: (1) Prospective and retrospective studies; (2) Studies published in English language; (3) Patients diagnosed with VRL or IOL; (4) Various treatment options, such as monotherapy or MTX-based first-line therapy; (5) Studies reporting extractable endpoints, including the overall response rate (ORR), complete response (CR), partial response (PR), survival data, and adverse events (AEs). Meanwhile, studies meeting any of the following criteria were excluded from this review: (1) Duplicate literature; (2) Reviews, case reports, and cellular or animal studies; (3) Non-therapeutic or diagnostic studies; (4) Studies from which data could not be extracted; (5) Updates of previous results; (6) Lymphoma with primary site in the ciliary body or choroid; (7) Articles published earlier than 2010.

The study selection process can be broadly divided into two stages. First, two investigators (Jing Gao, Lang Wang) independently evaluated the title and abstract of each article to determine its eligibility for inclusion in the meta-analysis. Subsequently, the two investigators compared the full text of the studies that met the criteria established in the first stage, with any discrepancies resolved through discussion or consultation with a third researcher (Xiaoyan Peng).

### Data analysis

The data collection process from eligible studies was conducted independently by two authors, with any discrepancies being resolved through joint discussion with a third author. An Excel sheet was utilized to extract information from the studies, which included the name of the first author, publication year, country, study period, study design (type of study and trial phase), median follow-up time, disease status, sample size, median age, patient gender, primary intervention, and main outcomes (response, survival, and AEs).

The ORRs, CRRs, and 3/4 AEs from the included literature were analyzed and combined using forest plots. The survival data units were inconsistent among the included literature, and only five studies had results for median progression-free survival (mPFS) [6–10]. Therefore, we pooled extractable survival data from the literature and analyzed them using the Kaplan–Meier method and the log-rank test in order to draw survival curves, make survival comparisons, and calculate mPFS for each group. The Engauge digitizing software version 10.8 was used to obtain a portion of the survival data from Kaplan–Meier curves.

Heterogeneity across studies was assessed using Cochran's Q test and I2 statistics. A fixed effects model was used for data combination when heterogeneity was not significant (I2 < 50% or *p*-value > 0.1), and a random effects model was used when heterogeneity was significant. Egger's test was utilized to investigate publication bias, with *P*-values indicating the significance of bias and *P* < 0.05 indicating a significant publication bias. All statistical analyses were performed using R version 4.1.1 (R Foundation for Statistical Computing, Vienna, Austria).

## Results

### Study selection

**Fig. 1:**
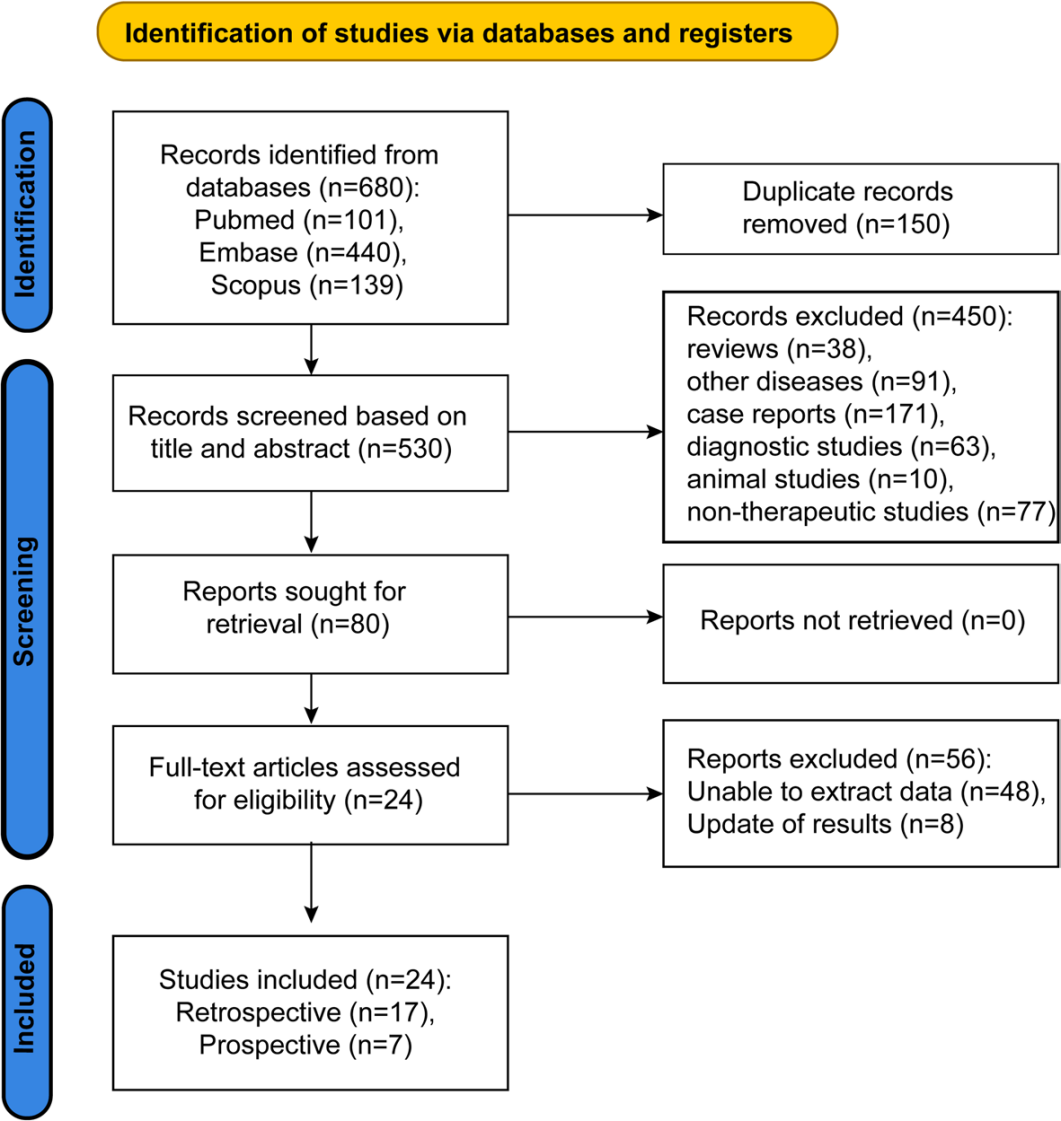
Flow diagram of study selection

Upon an initial search, 680 pertinent records were acquired. After eliminating 150 duplicates, we meticulously examined the titles and abstracts of the remaining 530 publications. Out of these, 450 studies were disregarded for failing to meet the eligibility criteria: reviews (*n* = 38), research on different diseases (*n* = 91), case reports (*n* = 171), diagnostic studies (*n* = 63), animal studies (*n* = 10), and non-therapeutic studies (*n* = 77). From the remaining 80 records, the complete text was scrutinized and 56 of them were dismissed due to the following reasons: inability to extract data (*n* = 48), and update of results (*n* = 8). Eventually, 24 full-text articles or conference abstracts qualified for assessment, which comprised of 17 retrospective studies [3, 4, 6–20] and 7 prospective studies [21–27]. The specific studies screening process is depicted in Fig. 1.

### Study characteristicsm

Out of the aforementioned 24 studies, 19 studies received first-line combination regimens, which included intraocular MTX injections, systemic high-dose MTX chemotherapy, local radiotherapy, and other targeted therapy regimens such as Lenalidomide and BTKi. Meanwhile, 5 studies utilized monotherapy, including the administration of pembrolizumab, BTKi, and temozolomide. A total of 517 patients with vitreoretinal lymphoma were included in these studies, with 453 having gender data, out of which 183 were male and 270 were female. The gender information of 64 patients was missing. The age range of the patients was 31–90 years, with a median age of 64 years. The follow-up duration of all studies ranged from 0.2–246 months, with a median follow-up time of 30.03 months. Five of the included studies had a median follow-up time of less than 24 months (Two BTKi alone [4, 25], two ioMTX in combination with lenalidomide/BTKi [24, 27] and one using multiple combination therapies [16]). The patient characteristics of the studies included are showed in Table 1.

**Table 1:** Baseline clinical characteristics of included studies

Study	Year	Country	Design	Study period	Median follow-up time, months (range)	Disease status	Sample size	Median age, years (range)	Gender male/female	Primary intervention	Main outcomes
de la Fuente, et al	2019	United States	Retrospective	2005/2–2018/8	68(17–154)	Primary vitreoretinal lymphoma (PVRL)	12	64(38–81)	7/5	Bilateral RT + MTX-based chemotherapy	CR, OR, PFS, OS, AEs
Kaburaki, et al	2017	Japan	One-arm Prospective Trial	2008/8–2015/3	48.9(15.3–95.1)	Primary intraocular lymphoma (PIOL)	17	63(43–72)	9/8	ioMTX + R-MPV + rdWBRT	CR, OR, PFS, OS, AEs
Hoang-Xuan, et al	2020	France	Prospective Multi-center, Open-label, Phase II trial	2017/7–2019/10	6.7(0.2–27.4)	Primary CNS lymphoma (PCNSL) and PVRL	50(9)	72(43–83)	/	Pembrolizumab monotherapy	CR, OR, PFS, AEs
Akiyama, et al	2016	Japan	Single-arm Prospective study	2007/1–2013/12	29.5	PIOL	10	68.5	4/6	ioMTX + systemic high-dose MTX	CR, OR, AEs
Taoka, et al	2012	Japan	Retrospective	2007/11–2009/12	32(21–42)	PIOL	5	65(43–72)	2/3	ioMTX + R-MPV + rdWBRT	DFS, CR
Soussain, et al	2019	France	Prospective Multi-center, Open-label, Phase II trial	2015/9–2016/7	25.7(0.7–30.5)	R/R PCNSL and PVRL	44(14)	70(52–81)	/	Ibrutinib	CR, OR, OS, PFS, AEs
Zhang, et al	2022	China	Prospective Multi-center, Open-label, Phase II trial	2020/8–2022/1	12.4(0.3–18.1)	PVRL	10	55(39–70)	3/7	Btki + ioMTX	PFS, AEs
Guan, et al	2022	China	Prospective Single-center, Open-label, Phase II trial	2020/10–2022/4	8.3(2.5–21.4)	Vitreoretinal lymphoma (VRL)	10	/	/	Ibrutinib zanubrutinib orelabrutinib	CR, OR, PFS, OS
Zhang, et al	2021	China	Prospective Single-center, Open-label, Phase II trial	2018/8–2020/1	18.3(10.6–27.8)	PVRL	11	58(48–70)	3/8	R2 + ioMTX Lenalidomide maintain	CR, OR, PFS, OS
Baron, et al	2020	France	Retrospective	/	42(9–115)	PVRL	21	75(35–90)	/	Temozolomide	CR, OR, PFS, OS
Zhou, et al	2022	China	Retrospective	2009/4–2019/8	30.55(12–73)	VRL	40	62.5(31–81)	14/26	ioMTX + MTX-based chemotherapy	mPFS, OS
Anthony, et al	2021	United States	Retrospective	/	26(3–49)	PVRL	7	69(56–85)	4/3	ioMTX ± systemic chemotherapy	CR, OR, PFS
Hsu, et al	2022	China	Retrospective	2013/1–2018/1	/	Intraocular lymphoma (IOL)	12	/	5/7	ioMTX + systemic high-dose MTX	CR, OR, OS, PFS, AEs
Ma, et al	2016	China	Retrospective	2003/1–2013/12	40.2(4.4–123.3)	PIOL	19	57(39–77)	6/13	ioMTX + systemic high-dose MTX	CR, OR, OS, mPFS, AEs
Lam, et al	2021	French	Retrospective	2011/1–2018/3	61(50–71)	PVRL	59	70(39–88)	14/45	IV HD-MTX based systemic therapy	CR, OR, mPFS, OS, AEs
Wang, et al	2021	China	Retrospective	2020/5-?	7.5(4–15)	VRL	11	61(41–73)	4/7	zanubrutinib orelabrutin	CR, OR, PFS, AEs
Cheah, et al	2016	United States	Retrospective database	2007/10–2015/4	50.4(21.6–91.2)	Primary intraocular lymphoma (PIOL)	11	66(48–72)	2/9	Bilateral RT + MTX-based chemotherapy	CR, OR, AEs, mPFS, OS
Klimova, et al	2018	Czech Republic	Retrospective	2004–2016	56(3–166)	PVRL and PCNSL	20 (10)	20 (10)	/	multiple combination therapies	5OS, PFS, OS
Castellino, et al	2019	United States	Retrospective	1990–2018	33.6(1.2–175.2)	primary and concurrent VRL	69	65(36–85)	34/35	multiple combination therapies	mFFS, CNS-RFS,OS
Gozzi, et al	2021	Italy	Retrospective	2006/1–2020/10	22(9–58)	VRL	22	65(55–72)	10/12	multiple combination therapies	5OS, PFS
Kim, et al	2016	United States	Retrospective	1994–2010	29(10.2–96.4)	primary and concurrent IOL	22	65	8/14	multiple combination therapies	FFS, OS
Riemens, et al	2015	Europe	Retrospective	1991/1–2012/12	49(15–246)	primary vitreoretinal lymphoma (PVRL)	78	58(38–86)	34/44	multiple combination therapies	OS, PFS
Lee, et al	2015	Korea	Retrospective	2007/12–2014/6	/	IOL	20	59(34–76)	13/7	multiple combination therapies	mPFS, OS
Teckie, et al	2014	United States	Retrospective	1999–2011	25(2–150)	primary intraocular lymphoma (PIOL)	18	64(32–82)	7/11	multiple combination therapies	ORR, 2OS, 2PFS

*Abbreviations*: *PVRL* Primary vitreoretinal lymphoma, *PIOL* Primary intraocular lymphoma, *PCNSL* Primary central nervous system lymphoma, *VRL* Vitreoretinal lymphoma, *MTX* Methotrexate, *ioMTX* intravitreal MTX, *R-MPV* Rituximab, methotrexate, procarbazine, vincristine, *rdWBRT* reduced-dose whole-brain radiotherapy, *Btki* Bruton tyrosine kinase inhibitors, *OR* Overall response, *CR* Complete response, *OS* Overall survival, *mPFS* median progression-free survival, *AEs* Adverse event, *DFS* Disease-free survival

### Quality assessment

Since all 7 prospective studies were single-arm trials, we employed the Risk of Bias in Non-Randomized Studies-of Intervention (ROBINS-I) tool to evaluate their risk [28]. The remaining 17 retrospective studies were assessed for risk using the JBI Critical Appraisal Checklist for Case Series. Out of the 7 single-arm trials, 3 were deemed to have low or moderate bias overall, while 4 were found to have serious bias. Among the 17 retrospective studies, 1 study had 2 questions that did not meet the criteria, 3 had 1 question that did not meet the criteria, and 14 met all the criteria for the questions. The final risk assessment outcomes are summarized in Supplementary Table 2.

### Efficacy

#### Tumor response

**Fig. 2:**
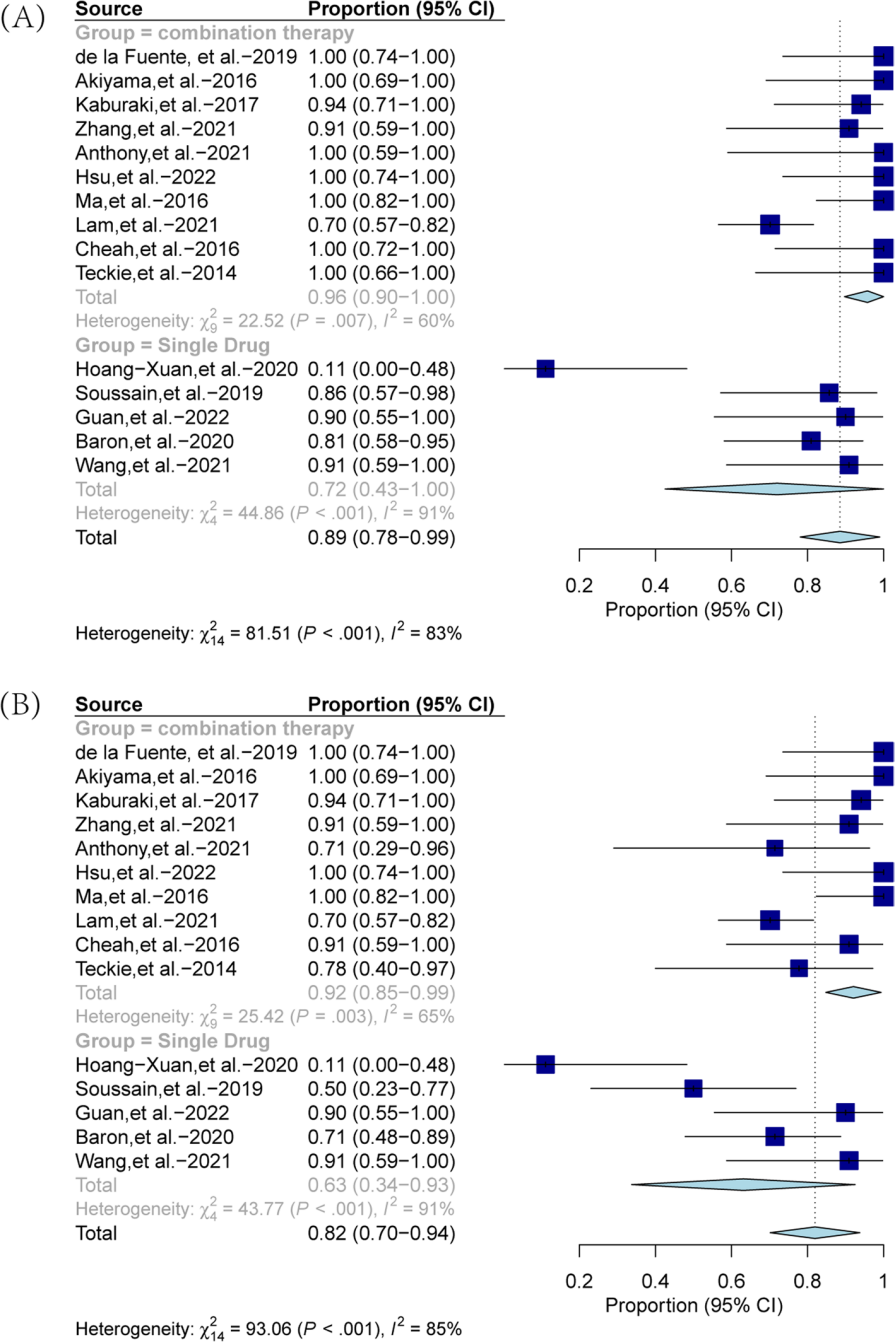
Forest plot for pooled overall response rate (**A**) and complete response rate (**B**) across the combined treatment group and the monotherapy group

A total of 15 publications reported complete response (CR), partial response (PR), as well as overall response (CR + PR) to measure the tumor response to treatment. CR is defined as the patient achieving symptom remission after treatment, having no residual lesions in the anterior chamber, vitreous body, or retina, and returning to normal IL-10 levels. In contrast, PR is defined as partial remission of the disease after treatment, as evidenced by mild anterior chamber, vitreous or retinal lesions. The pooled ORR was 89% (95% CI, 0.78 to 0.99) for the entire cohort, and 96% (95% CI, 0.90 to 1.00) and 72% (95% CI, 0.43 to 1.00) for the combination and monotherapy groups, respectively. In addition, the pooled CRR for the entire cohort was 82% (95% CI, 0.70 to 0.94), while the pooled CRR for the combination and monotherapy groups was 92% (95% CI, 0.85 to 0.99) and 63% (95% CI, 0.34 to 0.93), respectively. The forest plots depicting these results are shown in Fig. 2.

**Fig. 3:**
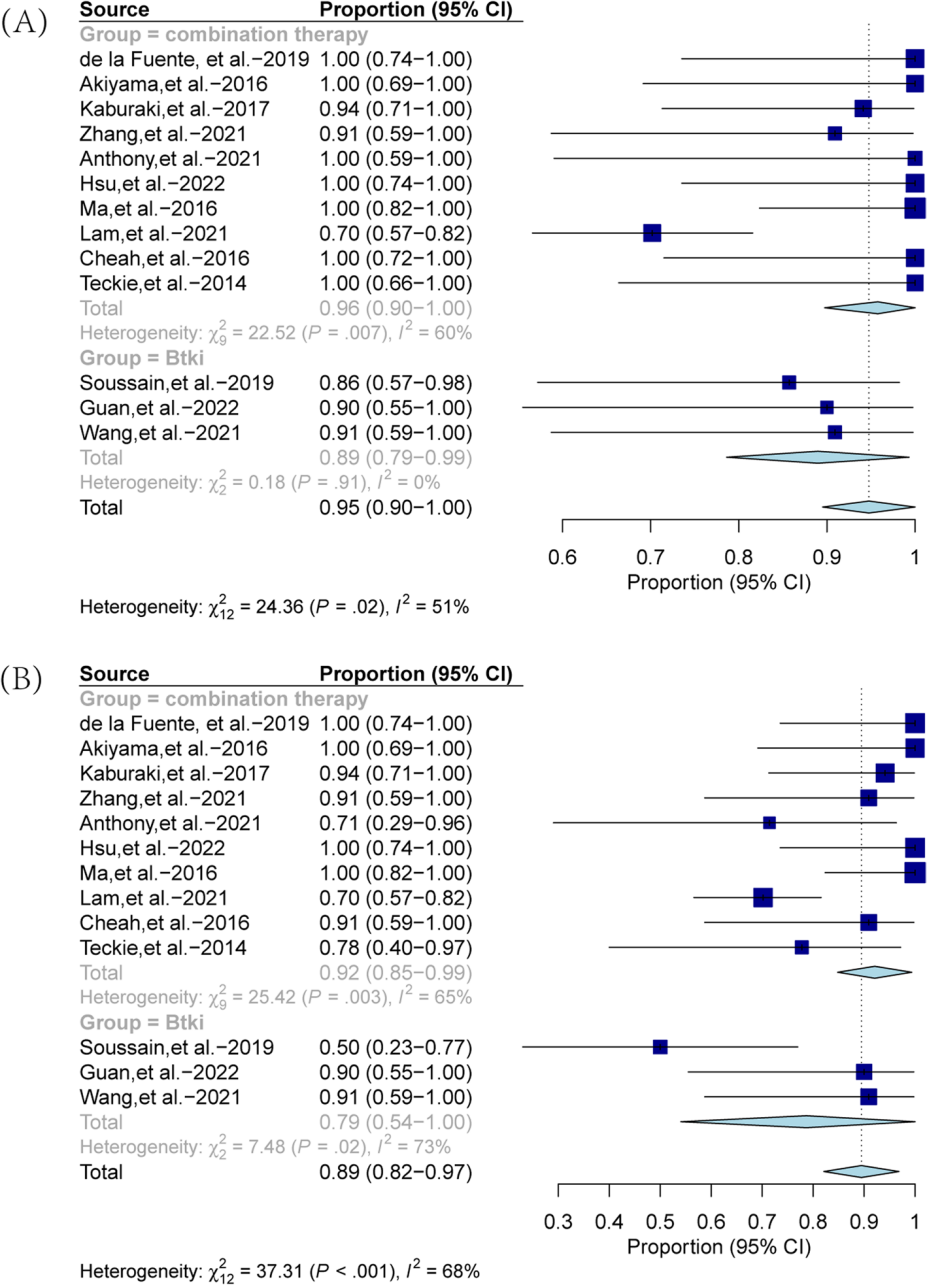
Forest plot for pooled overall response rate (**A**) and complete response rate (**B**) across the combined treatment group and the BTKi group

We have also conducted a comparative analysis of the pooled ORR and CRR between the combination treatment group and the BTKi monotherapy group. Figure 3 showed that the combination treatment group demonstrated a pooled ORR comparable to that of the BTKi group, with values of 96% (95% CI, 0.90–1.00) and 89% (95% CI, 0.79–0.99), respectively. However, the CRR of the combination treatment group (pooled value of 92%, 95% CI: 0.85–0.99) was higher than that of the BTKi group (pooled value of 79%, 95% CI: 0.54–1.00). We further performed subgroup analyses on the monotherapy treatment group and found that among the five studies, three employed BTK inhibitors while the other two used different single drugs. Forest plots of combined ORR and CRR rates for the two groups are presented in Figure S1 and S2 to compare the efficacy of BTK inhibitors with other single drugs. The results indicated that the combined ORR value of the BTK inhibitor group is significantly higher than that of the non-BTK inhibitor group. This finding suggests that BTK inhibitors may be a promising treatment option for vitreoretinal lymphoma.

#### Survival

**Fig. 4:**
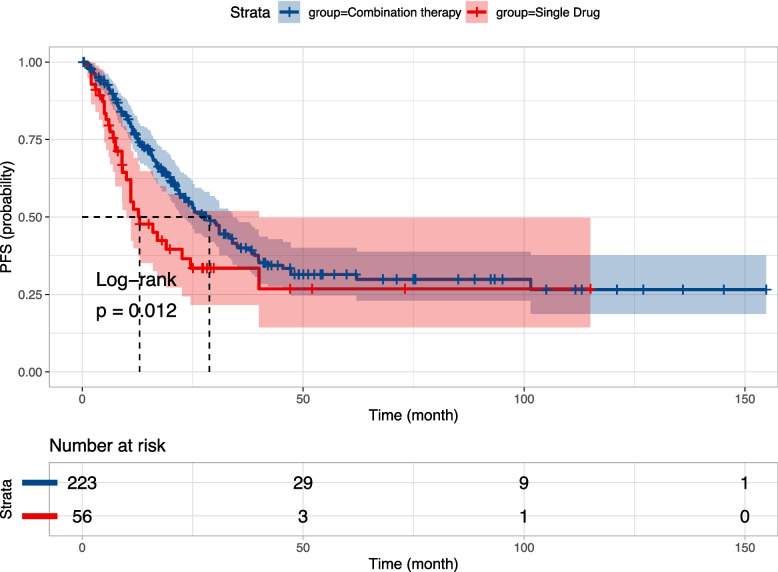
Survival curves in the combined treatment group versus the monotherapy group

**Fig. 5:**
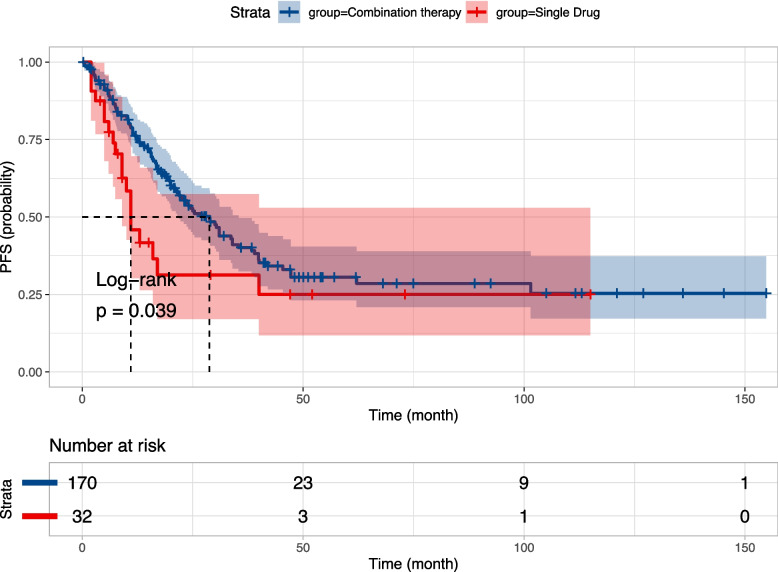
Survival curves in the combined treatment group versus the monotherapy group in the retrospective study subgroup

Seven prospective studies and twelve retrospective studies provided extractable survival data tables or K-M survival curves for a total of 279 patients following data extraction. Figure 4 displays the survival curves for the combined mPFS of 28.8 months (95% CI, 23 to 34) and 13 months (95% CI, 10.0 to 40) in the combined treatment group (*n* = 223) versus the monotherapy group (*n* = 56) from a total of 17 publications, respectively, demonstrating a significant difference (*p* = 0.012). In a subgroup analysis based on the retrospective article, the median progression-free survival was 28.8 months (95% CI, 21.9 to 35.4) in the combination treatment group (*n* = 170) compared to 11 months (95% CI, 9.0 to NA) in the combined monotherapy group (*n* = 32) in twelve retrospective studies, demonstrating a significant difference (*p* = 0.039), with the survival curves depicted in Fig. 5. Meanwhile, Figure S3 demonstrates a combined mPFS of 31 months (95% CI, 21 to NA) and 19 months (95% CI, 9.1 to NA) in the combination (*n* = 53) and monotherapy groups (*n* = 24), respectively, in the seven prospective studies.

Furthermore, four publications were included in the monotherapy group with treatments comprising temozolomide monotherapy and BTK inhibitors, and the two groups did not exhibit a significant difference in mPFS (*p* = 0.58) (Figure S4). In the combination treatment group, there was no significant survival difference between the three-approach combination regimen and the two-approach combination regimen or the ioMTX-based regimen, while age, gender, and whether or not bilateral eye onset were not found to be associated with survival time (Figure S5-S8). In contrast, the analysis revealed that targeted-agent such as Lenalidomide and BTKi combined with ioMTX therapy had poorer survival outcomes compared to other combination therapies (Figure S9). This implies that systemic chemotherapy regimens based on MTX may be more efficacious when used in conjunction with ioMTX as opposed to lenalidomide and BTKi.

### Toxicity and relapse

**Fig. 6:**
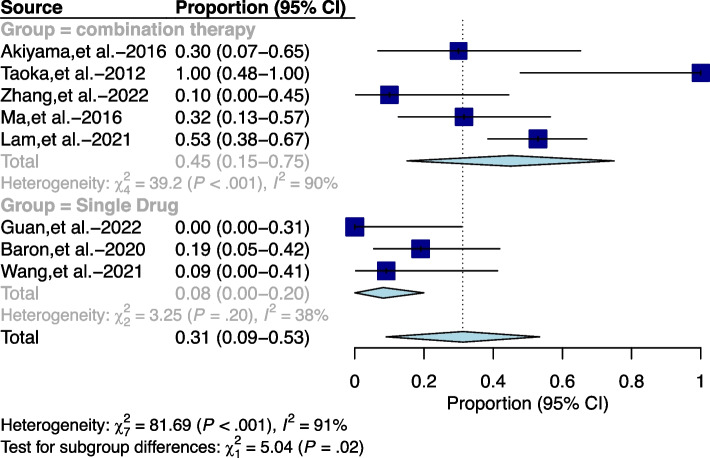
Forest plot for pooled grade 3/4 adverse events (AEs) across the combined treatment group and the monotherapy group

Eight publications reported grade 3/4 adverse events (AEs), consisting mainly of cataract, neutropenia, anemia, and hepatic and renal toxicity. In the overall analysis, the pooled grade 3/4 toxicity was 45% (95% CI: 0.15–0.75) for the combination therapy group compared to 8% (95% CI: 0.0–0.20) for the monotherapy group, and the overall pooled value was 31% (95% CI: 0.09–0.53), indicating that less severe toxicity occurred in the monotherapy group than in the combination therapy group, as depicted in Fig. 6. Moreover, in the combination treatment group, major serious toxic reactions included cataract (pooled value of 43%, 95% CI: 0.26–0.61) and neutropenia (pooled value of 35%, 95% CI: 0.00–0.77), as illustrated in Figure S10 and S11. While in the monotherapy group, only four cases of grade 3/4 toxicity were reported in the literature using temozolomide, which consisted of 3 cases of grade 3 anemia and vomiting and 1 case of grade 4 neutropenia and thrombocytopenia.

The forest plot in Figure S12 shows that central nervous system (CNS) relapse was reported in 14 publications in the combination therapy group, while only 1 publication reported it in the monotherapy group. The incidence rate of CNS relapse was 31% (95% CI: 0.25–0.37) in the combination therapy group, with a median follow-up time of 33.6 months (ranging from 0.3 to 175.2 months). In the monotherapy group, only one trial provided information on CNS relapse, with a 30% incidence and a median follow-up time of 8.3 months (ranging from 2.5 to 21.4 months). Twelve publications in the combination therapy group reported ocular recurrence with a combined incidence of 24% (95% CI, 0.17 to 0.31) (Figure S13), while one publication in the monotherapy group reported ocular recurrence in 2 of 10 patients [25]. However, due to the limited number of studies and small sample size in the monotherapy group, further research is needed to better understand any potential differences in ocular recurrence between the two treatment approaches.

## Discussion

Intraocular lymphoma, being an uncommon ailment, can be mistakenly identified as uveitis during diagnosis. Additionally, a treatment approach that achieves both effectiveness and safety remains elusive. Moreover, the long-term prognosis for PVRL patients is bleak, as around 60%-80% of them eventually develop PCNSL, as reported in 1999 by Akpek [29]. Treatment modalities for PVRL are variable and include methotrexate-based local/systemic therapy, local/whole-brain radiation therapy, various monotherapy and intensive chemotherapy plus hematopoietic stem cell transplantation (IC + ASCT), the optimal treatment modality has not yet been identified.

Intravitreal methotrexate (ioMTX) is an early proposed local treatment with a high remission rate but usually a poor prognosis, with most patients experiencing CNS progression within a short period of time (2004 Coupland) [30]. Anthony et al. designed a small sample single-center retrospective study to investigate the efficacy of ioMTX alone in the treatment of PVRL [11]. Although all achieved CR or PR, their time until disease recurrence was not promising (mean time to first recurrence was 6.5 months). Based on the anatomical and functional similarities of PVRL to PCNSL at the blood–brain barrier (BBB) and blood-retinal barrier (BRB), intravenous high-dose methotrexate injection (IV HD-MTX) has also been used as an empirical agent for PVRL treatment and is often combined with ioMTX and local radiotherapy to improve efficacy (Akiyama, de la Fuente) [19, 22].

Lam et al. gathered data pertaining to 59 patients who were diagnosed with isolated primary vitreoretinal lymphoma (PVRL) from the French LOC network database, in order to examine the effectiveness and adverse effects of intravenous high-dose methotrexate (HD-MTX)-based systemic chemotherapy in treating PVRL patients [8]. Despite the fact that 70% of patients attained a complete response (CR) or unconfirmed complete response (uCR), the relapse rate was not insignificant (with a median follow-up of 61 months, 37% of patients experienced central nervous system recurrence and 58% had ocular recurrence), and the occurrence of grade 3/4 toxicity in 53% of patients implied poor tolerability. Furthermore, there are instances of using more than two regimens for treating PVRL. Kaburaki et al.'s R-MTX + ioMTX + rdWBRT (reduced-dose whole brain radiation therapy) regimen for PVRL resulted in long-term progression-free survival (PFS) and overall survival (OS) (with a 4-year PFS of 74.9% and a 4-year OS of 86.3%), as well as a low overall relapse rate (23.5%) [26]. However, it is important to note that this regimen was associated with significant grade 3/4 hematologic toxicity.

In addition, intraocular injection of rituximab as another intravitreal treatment for VRL, usually at a dose of 1 mg/0.1 mL, was first used by Kitzmann et al. in 2007 and achieved rapid remission in 8 eyes of 5 patients, demonstrating good tolerability [31]. Subsequently, Hashida et al. included 13 patients' 20 eyes in their 2012 study, all of whom exhibited severe corneal epithelial lesions or were unresponsive to repeated ioMTX injections. Following a course of intraocular rituximab therapy, initial control of retinal lesions was achieved. However, within three months, 11 eyes (55%) of nine patients experienced ocular relapse, and 9 cases (69%) eventually progressed to central nervous system involvement [32]. In 2014, Larkin et al. designed a multinational, multicenter study with a larger cohort, consisting of 48 eyes from 34 patients. The treatment was based on intravitreal injection of rituximab. Ultimately, complete and partial remission were observed in 31 eyes (64.6%) and 11 eyes (22.9%), respectively, while 12 eyes (25%) suffered complications, potentially due to rituximab [33]. To assess the therapeutic response and safety of intravitreal rituximab in PVRL patients, Kakkassery et al. conducted a study in 2021, enrolling 20 eyes from 15 consecutively diagnosed PVRL patients. No other intravitreal or systemic injections were given during treatment. Following therapy, there was a significant reduction in vitreous haze scores, marked improvement in best-corrected visual acuity, and no manifestation of severe adverse effects [34].

In recent times, various monotherapy regimens have been investigated to identify an effective and safe treatment combination. Temozolomide (TMZ), a second-generation alkylating agent that is well-tolerated, has been found to have good penetrative capacity into the central nervous system and cerebrospinal fluid (CSF) (Reni, 2007) [35]. Baron et al. conducted a retrospective study using TMZ for the treatment of PVRL, which produced encouraging results (with an ORR of 81%, mPFS of 12 months, and a central nervous system [CNS] relapse rate of 23.8%) [3]. Bruton tyrosine kinase (BTK) is a crucial mediator molecule in B-cell proliferation, and its inhibitors have the potential to serve as therapeutic agents in various B-cell malignancies. However, it is yet to be determined whether such inhibitors can offer therapeutic benefit to patients with primary vitreoretinal lymphoma (PVRL). To address this, Soussain et al. designed a multicenter, open-label phase II clinical trial aimed at evaluating the efficacy of Ibrutinib as a single agent in patients with both primary central nervous system lymphoma (PCNSL) and PVRL [23]. Of the 14 patients with PVRL included in the study, 86% achieved remission after 2 months of treatment, with a median progression-free survival (PFS) value of 22.7 months. Moreover, single-agent combination intraocular methotrexate (ioMTX) regimens have also been investigated. Zhang et al. sequentially tried a regimen of R2 (lenalidomide plus rituximab) + ioMTX induction, lenalidomide maintenance therapy, and ZR (zanubrutinib plus rituximab) + ioMTX to further investigate the optimal treatment strategy for PVRL [24, 27].

Moreover, a prospective study designed by Soussain et al. evaluating the feasibility of intensive chemotherapy (consisting of high-dose thiotepa, busulfan and cyclophosphamide) plus hematopoietic stem cell transplantation as a treatment modality for relapsed or refractory CNS lymphoma and intraocular lymphoma with an ultimate 3-year overall survival rate of 63.7%. demonstrating the benefit of IC + ASCT in patients with relapsed PVRL, but this modality is only indicated for younger relapsed patients younger than 60 years of age who are well tolerated, and its safety is difficult to guarantee in patients older than 60 years of age [36].

In this meta-analysis, we compared the efficacy and safety of combination therapy versus monotherapy regimens for the treatment of VRL. In the combination therapy group, specific interventions included ioMTX + chemotherapy (in 4 studies), RT + chemotherapy (in 2 studies), ioMTX/HD-MTX based regimen (in 2 studies), ioMTX + RT + chemotherapy (in 2 studies), ioMTX + lenalidomide/BTKi (in 2 studies) and combination of multiple therapies (in 7 studies). In the monotherapy group included in our study, interventions included Pomalidomide, Temozolomide, and BTKi.

We found that patients receiving combination therapy demonstrated a higher ORR and CRR as well as a relatively longer median progression-free survival (PFS) compared to those receiving monotherapy. BTKi, as a single agent, achieved an ORR that approximated that of the combination group, suggesting a strong potential for the treatment of VRL. The analysis also explored whether the number of treatment approaches in the combination group had an impact on survival time. Interestingly, the results showed that there were no significant survival differences between treatment regimens combining three approaches versus those combining two or less approaches. This suggests that the number of treatment approaches may not be the primary factor influencing survival time in patients receiving combination therapy for the condition under study. Moreover, this study investigated the potential influence of demographic and clinical characteristics on survival time, including age, gender, and whether the onset of the condition was bilateral. The study results indicated that none of these factors demonstrated a significant association with survival time. In further subgroup analysis, we found that ioMTX plus monotherapy did not show superior survival compared to other combination therapies, suggesting that MTX-based systemic chemotherapy regimens may be more effective when combined with ioMTX compared to lenalidomide and BTKi, but the data on ioMTX plus monotherapy is limited and requires more data to confirm its true benefits.

In addition, we also observed that the combination therapy group exhibited a higher incidence of grade 3/4 toxicities. Grade 3/4 toxicities mainly included ocular and systemic toxicities, with ocular toxicities mainly consisting of cataracts and keratitis, and systemic toxicities mainly consisting of neutropenia and anemia. We found that the combination therapy group had more significant grade 3/4 toxicities than the monotherapy group, suggesting that the safety of monotherapy may be better than that of combination therapy. Furthermore, we observed similar recurrence rates between the two groups. Whether it was a combination therapy or monotherapy, the CNS recurrence rate was approximately 30%. However, the ocular recurrence rate appeared to be relatively higher in the combination therapy group, but the number of studies included in the monotherapy treatment group was limited, and further research is needed to better understand the potential differences between the two treatment methods in terms of ocular recurrence.

In general, these results suggest that a treatment approach that combines efficacy and safety still needs to be explored to achieve better management of intraocular lymphoma. The long-term efficacy of systemic therapy with one drug alone is not satisfactory, and the combination of systemic and local therapy for intraocular lymphoma is the future trend. The use of single drugs such as BTK inhibitors and temozolomide in combination with high-dose systemic MTX chemotherapy regimens may also be a potential new research direction. Future research may focus on identifying the optimal treatment combination that can provide VRL patients with a longer period of remission, extended survival time, and prevention of recurrence in the central nervous system and ocular region.

The limitations of this article stem from the rarity of VRL, which results in a relatively small sample size. Additionally, the diverse treatment methods employed across various studies introduce significant heterogeneity, leading to some conclusions drawn from the combined data lacking statistical support. Moreover, the average follow-up time in the literature involving the use of new BTKi-like drugs is not yet long and may lead to some uncertainty in the results. Furthermore, the number of studies on oral monotherapy for VRL is small and more prospective studies are needed to facilitate a more robust comparison between the two groups. Nonetheless, our analysis provides a comprehensive summary of the efficacy and safety of various VRL treatment methods and serves as a valuable reference for further exploration of more optimal solutions.

## Conclusion

Based on the available evidence, first-line combination therapy for VRL appears to be more effective than monotherapy, with higher OR and CR rates and longer median progression-free survival. However, combination therapy also has higher rates of grade 3/4 toxicity compared to monotherapy. While BTK inhibitors as monotherapy for VRL appear to be well tolerated, further studies are needed to confirm their long-term efficacy. Prospective studies are necessary to evaluate the optimal treatment approach for VRL.

### Supplementary Information

**Additional file 1.**

**Additional file 2.**

**Additional file 3.**

**Additional file 4.**

**Additional file 5.**

**Additional file 6.**

**Additional file 7.**

**Additional file 8.**

**Additional file 9.**

**Additional file 10.**

**Additional file 11.**

**Additional file 12.**

**Additional file 13.**

**Additional file 14.**

**Additional file 15.**

## Data Availability

The datasets used and/or analysed during the current study are available from the corresponding author on reasonable request.
