# Supplementary figures and images for: Efficacy and safety of first-line combination therapy versus monotherapy for vitreoretinal lymphoma: a systematic review and meta-analysis
Source: BMC Ophthalmol. 2023 Nov 22;23:477. doi: 10.1186/s12886-023-03226-3 (PMC10664658; doi:10.1186/s12886-023-03226-3)

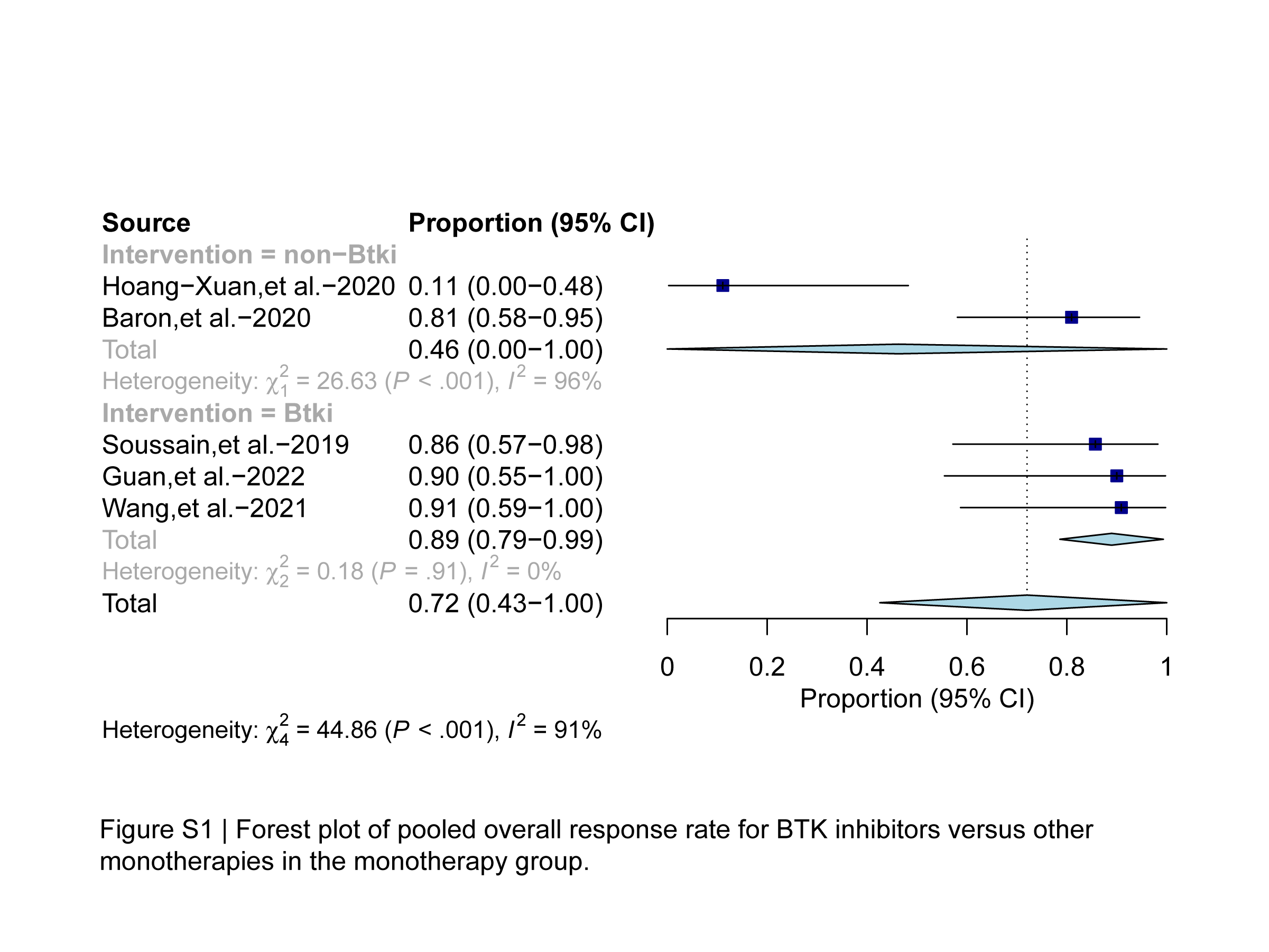

Supplement: Supplementary file 1 — Additional file 1. [file 12886_2023_3226_MOESM1_ESM.tif]

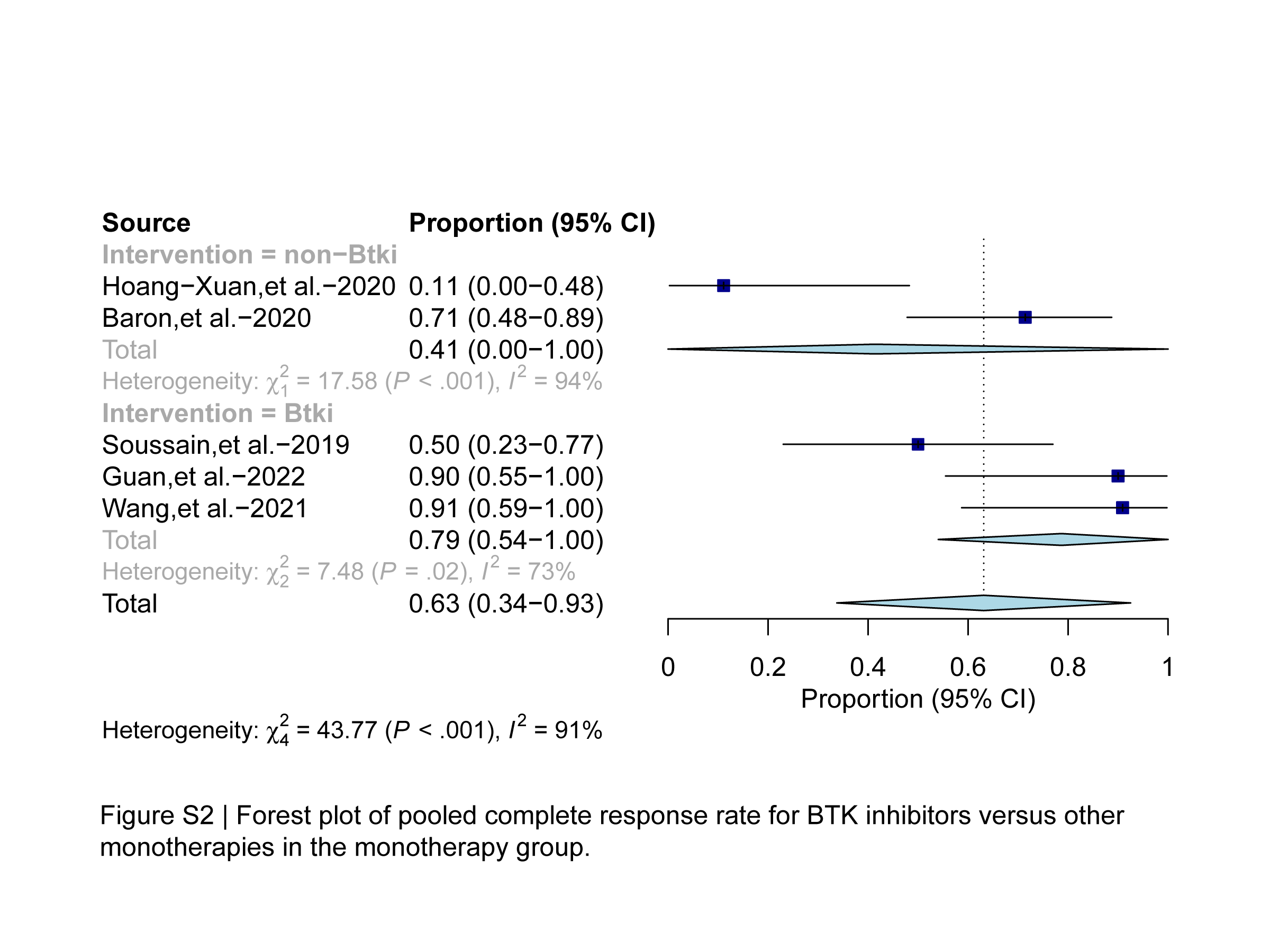

Supplement: Supplementary file 2 — Additional file 2. [file 12886_2023_3226_MOESM2_ESM.tif]

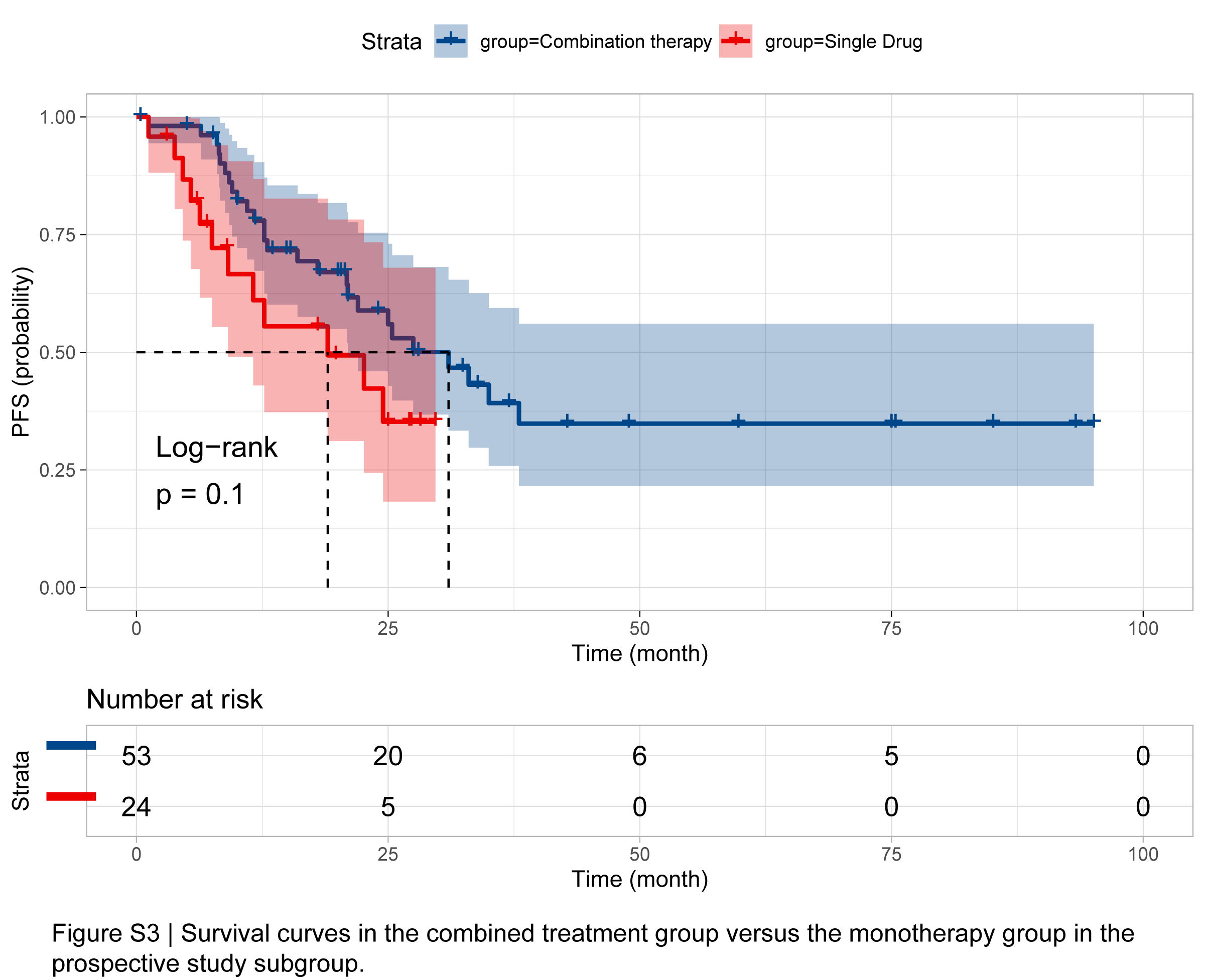

Supplement: Supplementary file 3 — Additional file 3. [file 12886_2023_3226_MOESM3_ESM.tif]

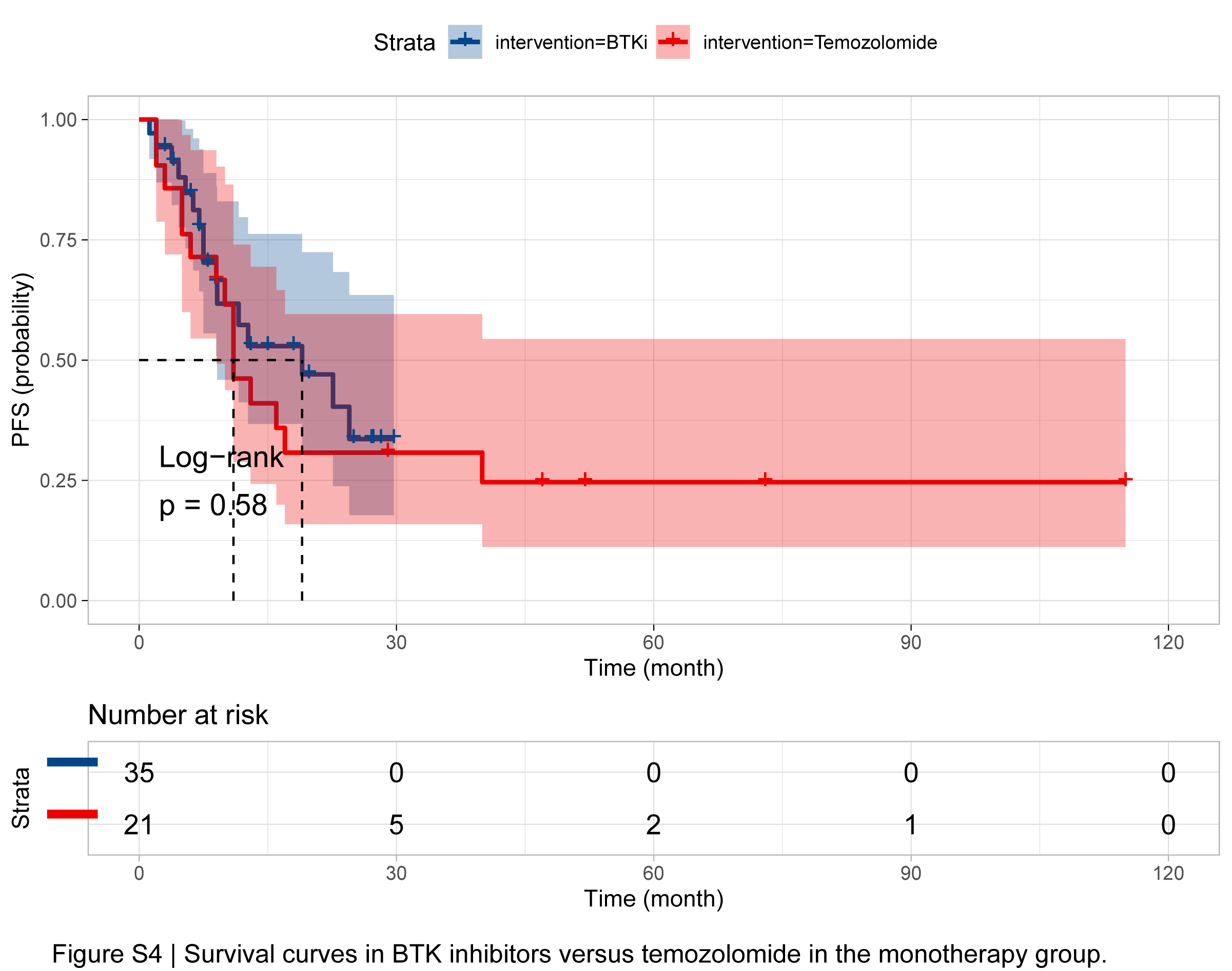

Supplement: Supplementary file 4 — Additional file 4. [file 12886_2023_3226_MOESM4_ESM.tif]

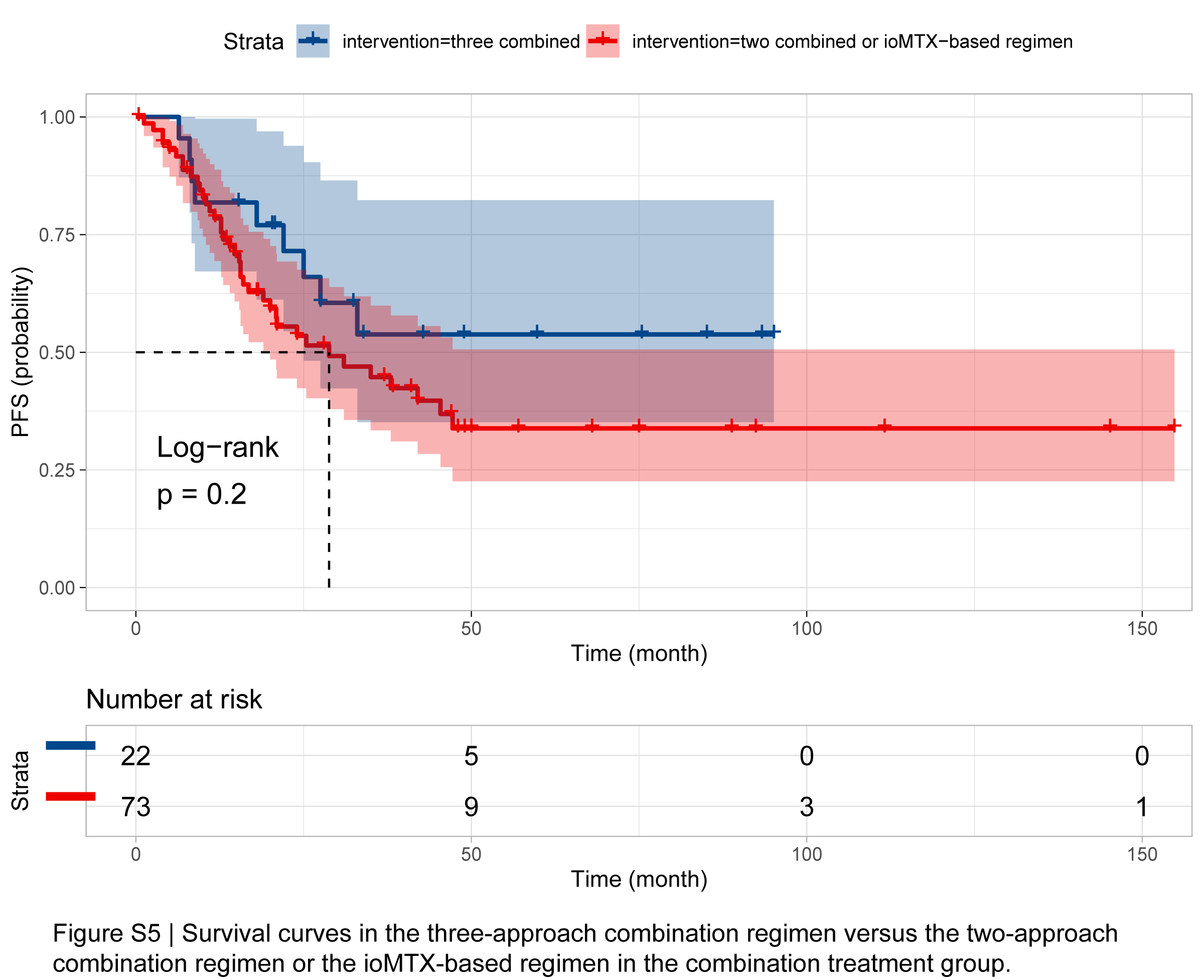

Supplement: Supplementary file 5 — Additional file 5. [file 12886_2023_3226_MOESM5_ESM.tif]

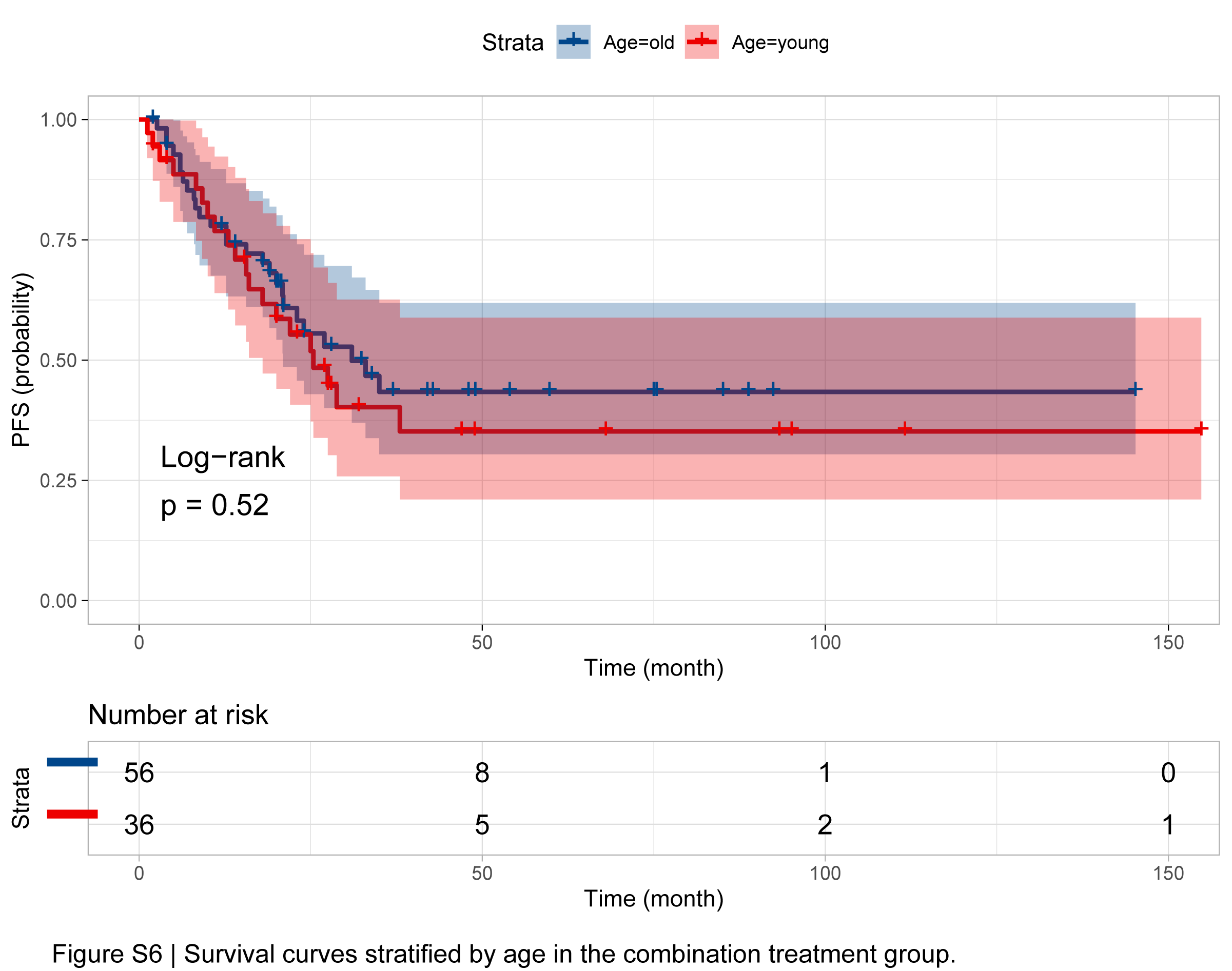

Supplement: Supplementary file 6 — Additional file 6. [file 12886_2023_3226_MOESM6_ESM.tif]

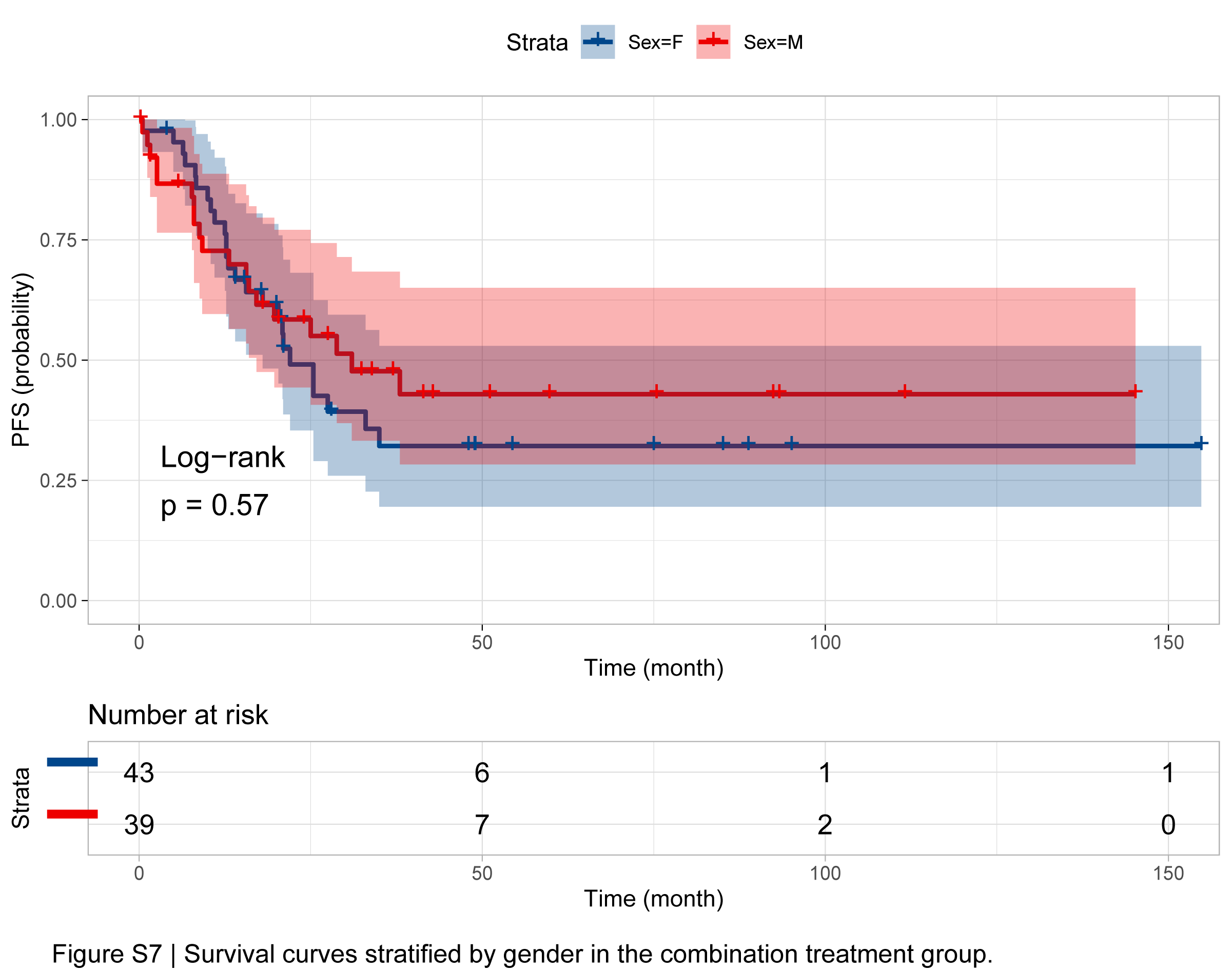

Supplement: Supplementary file 7 — Additional file 7. [file 12886_2023_3226_MOESM7_ESM.tif]

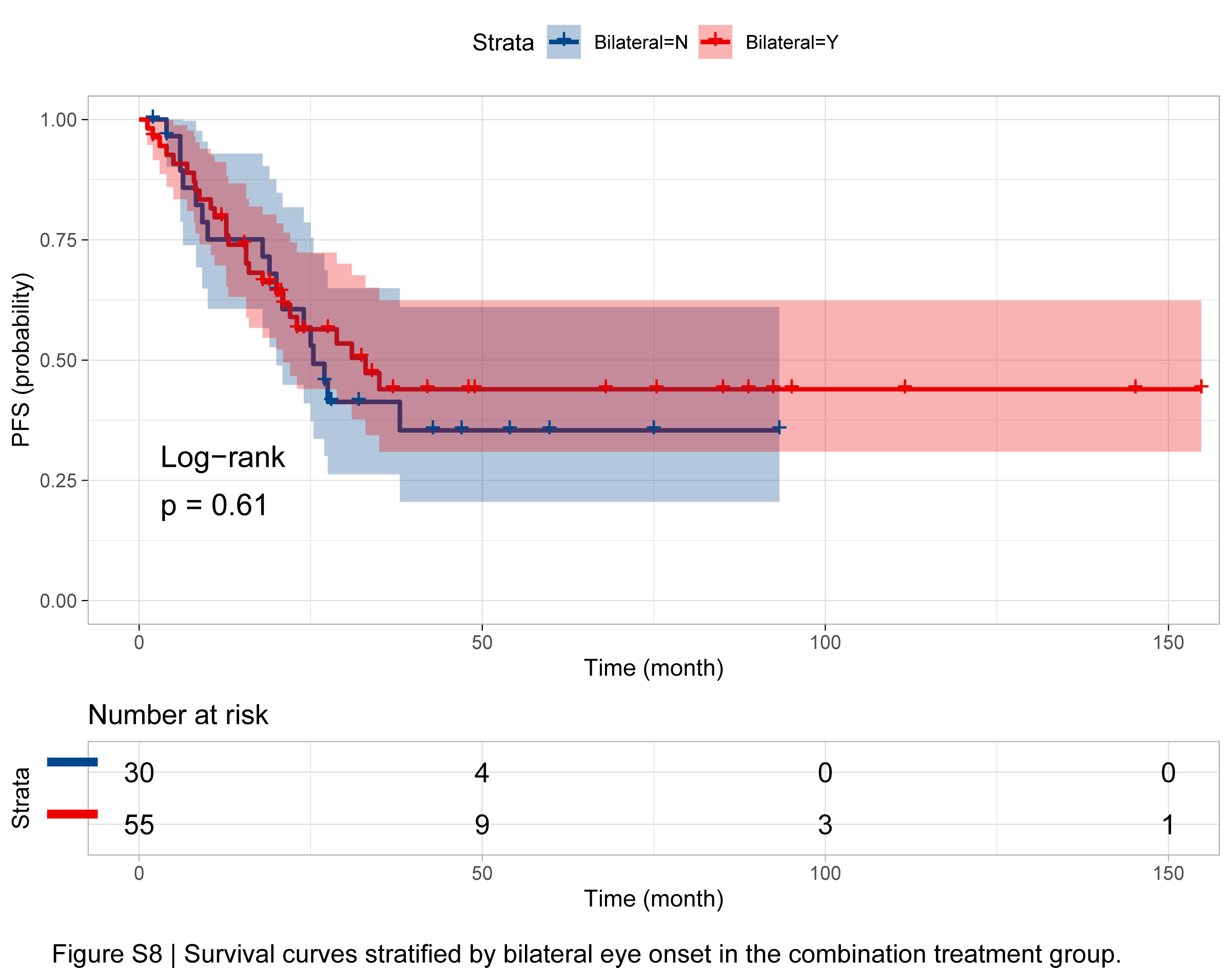

Supplement: Supplementary file 8 — Additional file 8. [file 12886_2023_3226_MOESM8_ESM.tif]

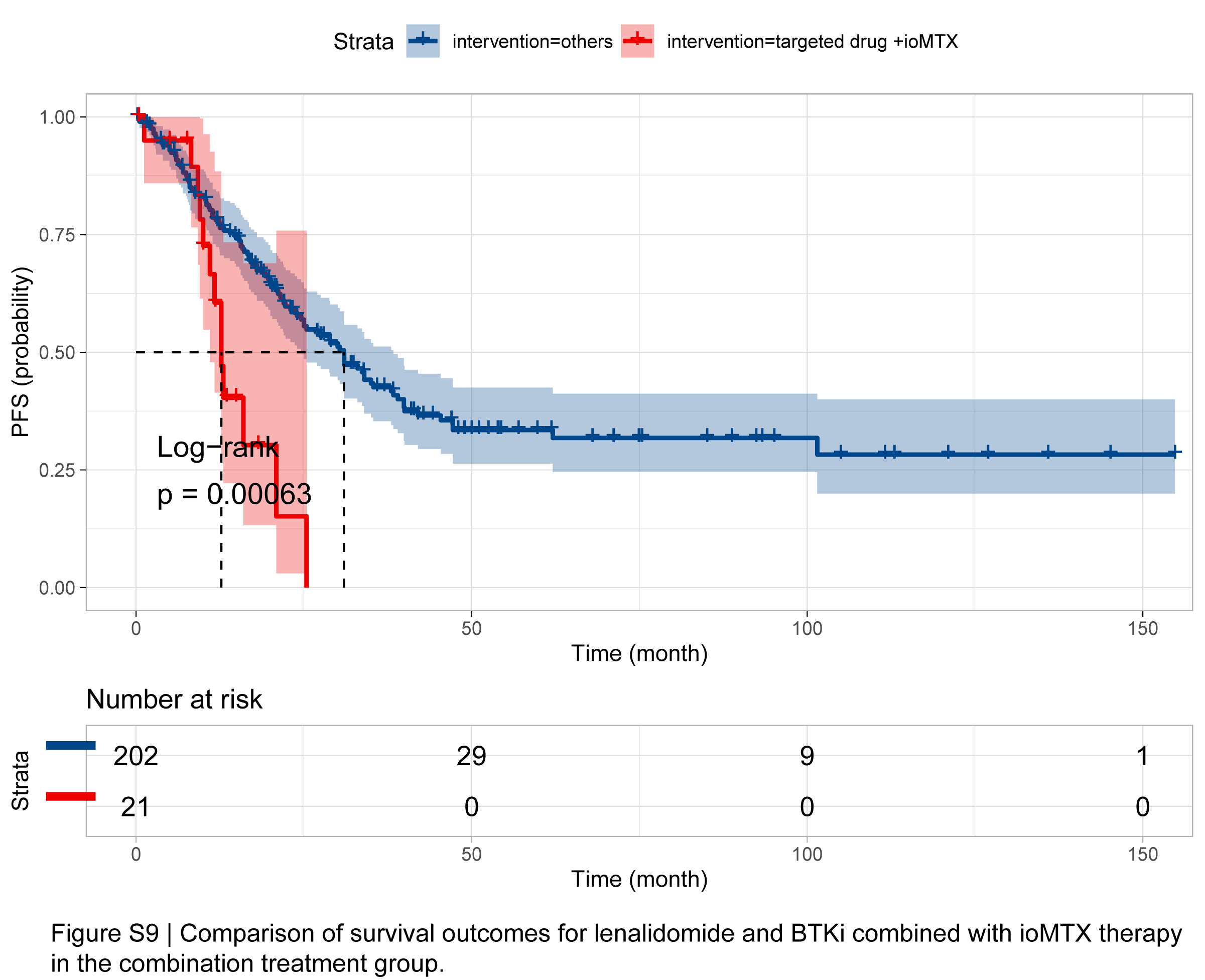

Supplement: Supplementary file 9 — Additional file 9. [file 12886_2023_3226_MOESM9_ESM.tif]

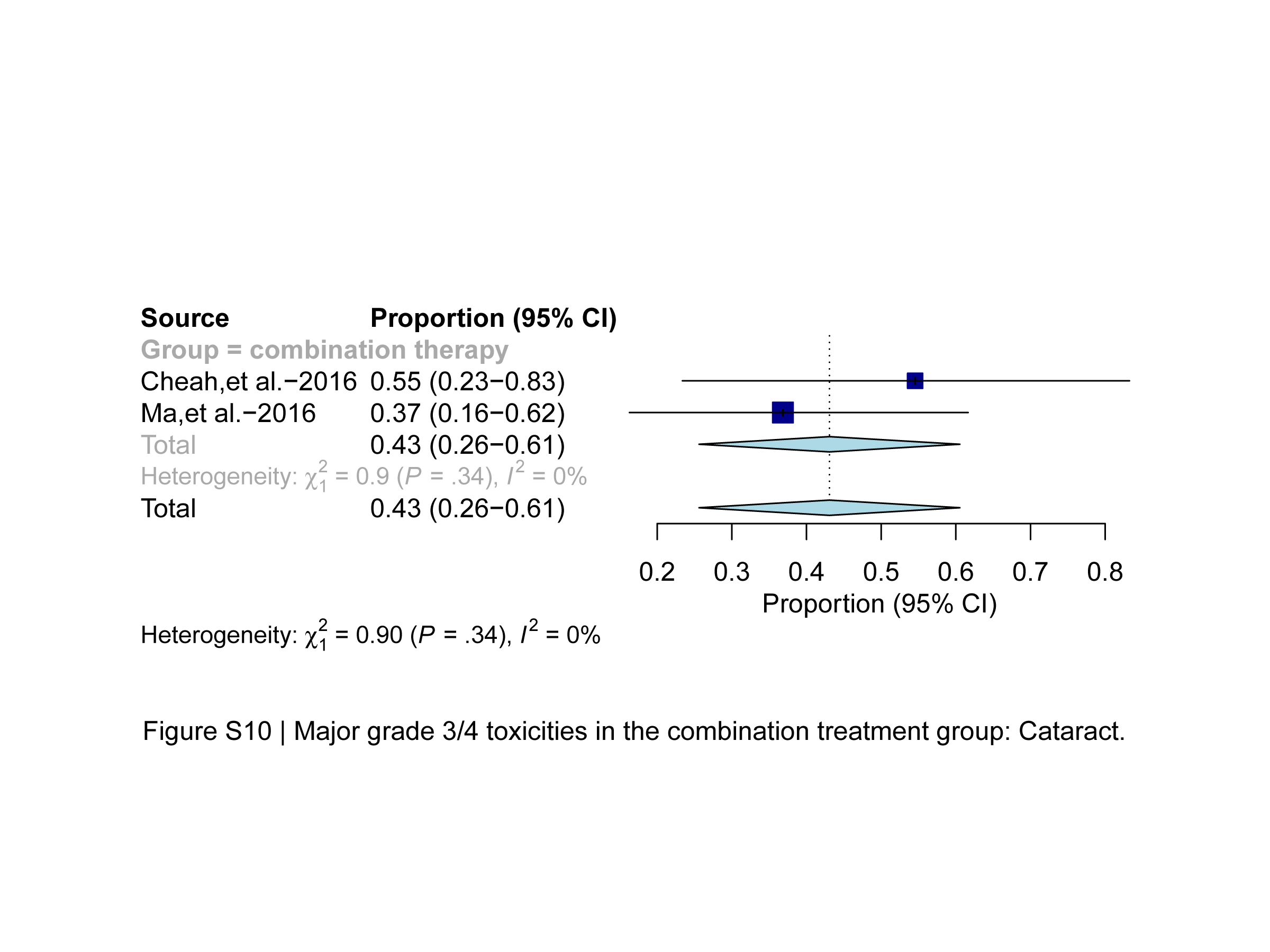

Supplement: Supplementary file 10 — Additional file 10. [file 12886_2023_3226_MOESM10_ESM.tif]

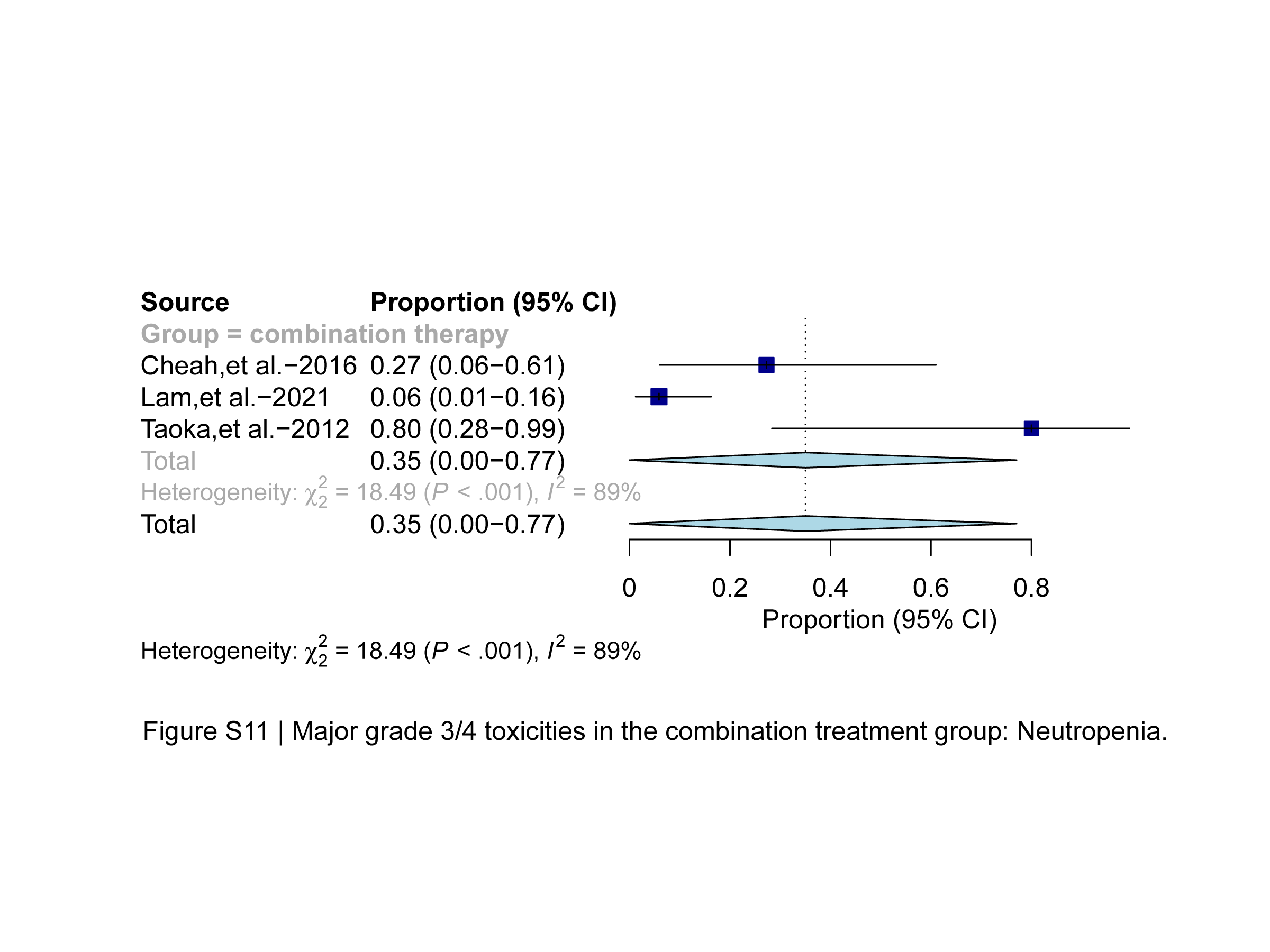

Supplement: Supplementary file 11 — Additional file 11. [file 12886_2023_3226_MOESM11_ESM.tif]

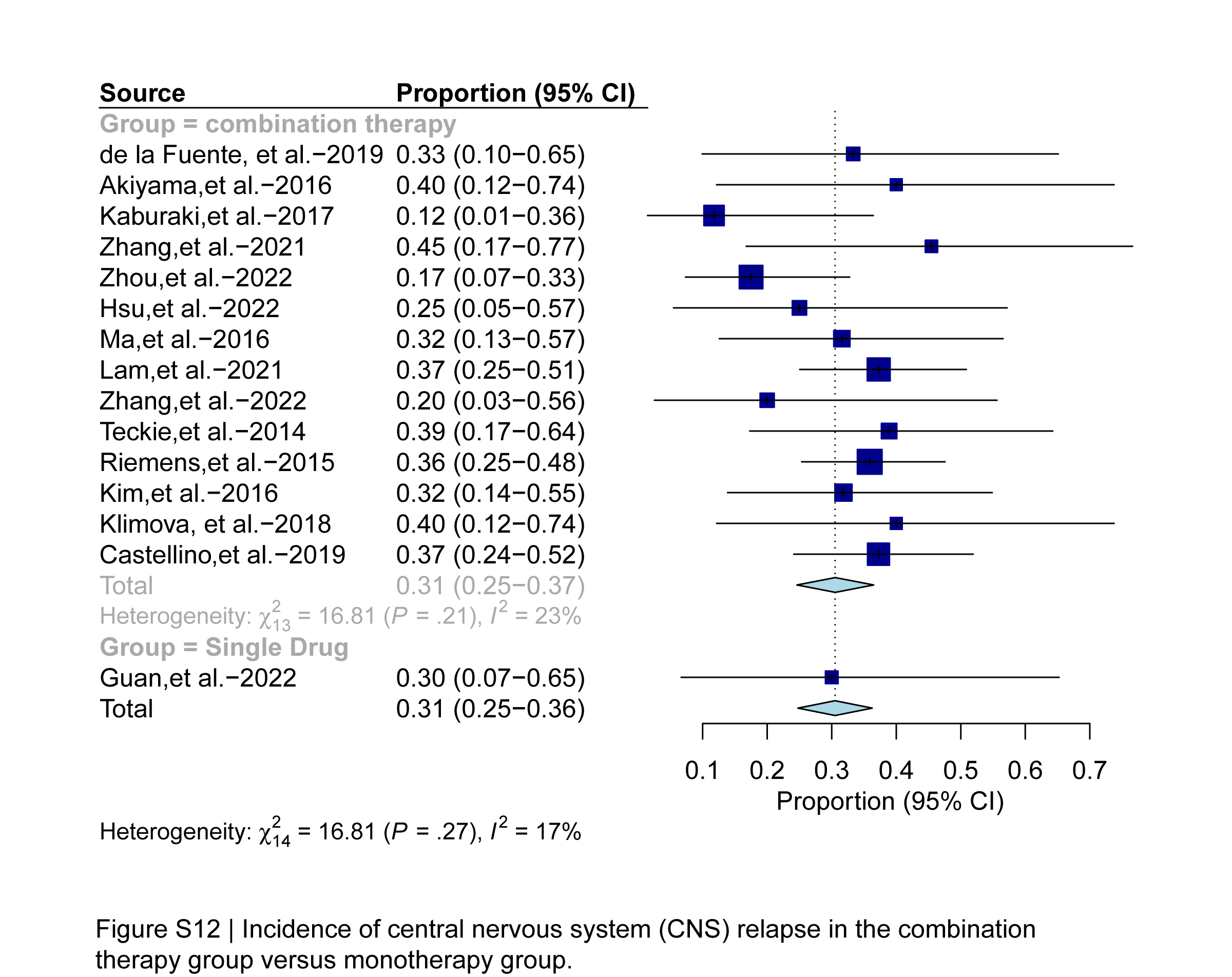

Supplement: Supplementary file 12 — Additional file 12. [file 12886_2023_3226_MOESM12_ESM.tif]

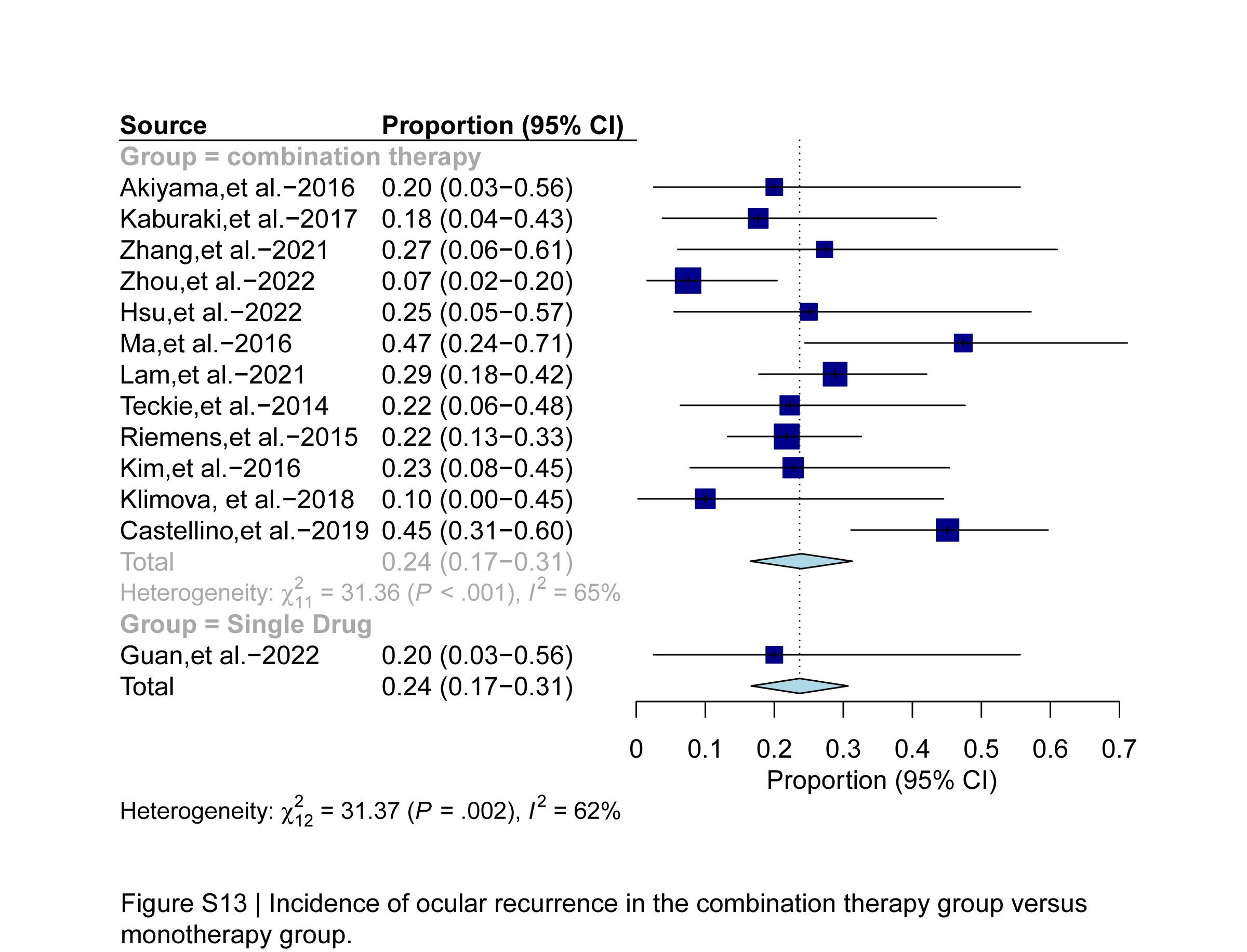

Supplement: Supplementary file 13 — Additional file 13. [file 12886_2023_3226_MOESM13_ESM.tif]
