# Supplementary material for: Efficacy and safety of first-line combination therapy versus monotherapy for vitreoretinal lymphoma: a systematic review and meta-analysis
Source: BMC Ophthalmol. 2023 Nov 22;23:477. doi: 10.1186/s12886-023-03226-3 (PMC10664658; doi:10.1186/s12886-023-03226-3)
Supplement: Supplementary file 15 — Additional file 15. [file 12886_2023_3226_MOESM15_ESM.docx]

**Supplementary Table 2** | Risk Assessment Outcomes for Included Studies.

| 1. **Risk of Bias in Non Randomized studies-of Intervention (ROBINS-I) tool for single-arm trials** | | | | | | | | |
| --- | --- | --- | --- | --- | --- | --- | --- | --- |
| **Study** | **Q1** | **Q2** | **Q3** | **Q4** | **Q5** | **Q6** | **Q7** | **overall appraisal** |
| Kaburaki,et al. | Low | Low | Low | Low | Low | Low | Low | Low |
| Hoang-Xuan,et al. | Low | Serious | Low | Critical | Low | Low | Critical | Critical |
| Akiyama,et al. | Low | Low | Low | Critical | Low | Low | Low | Critical |
| Soussain,et al. | Low | Low | Low | Moderate | Low | Low | Moderate | Moderate |
| Zhang,et al. (2022) | Low | Low | Low | No Information | Low | Low | Critical | Critical |
| Guan,et al. | Low | Serious | Low | Low | Low | Low | Low | Serious |
| Zhang,et al. (2021) | Low | Low | Low | Moderate | Low | Low | Moderate | Moderate |

Numbers for questions Q1–Q7 in heading refer to: 1) bias due to confounding, 2) bias in selection of participants into the study, 3) bias in classification of interventions, 4) bias due to deviations from intended interventions, 5) bias due to missing data, 6) bias in measurement of outcomes, and 7) bias in selection of the reported results.

| 1. **JBI Critical Appraisal Checklist for Case Series for included retrospective studies** | | | | | | | | | | | |
| --- | --- | --- | --- | --- | --- | --- | --- | --- | --- | --- | --- |
| **Study** | **Q1** | **Q2** | **Q3** | **Q4** | **Q5** | **Q6** | **Q7** | **Q8** | **Q9** | **Q10** | **overall appraisal** |
| Zhou,et al. | Yes | Yes | Yes | Yes | Yes | Yes | Yes | Yes | Yes | Yes | Inclued |
| Anthony,et al. | Yes | Yes | Yes | No | Yes | Yes | No | Yes | Yes | Yes | Inclued |
| Hsu,et al. | Yes | Yes | Yes | Yes | Yes | Yes | Yes | Yes | Yes | Yes | Inclued |
| Ma,et al. | Yes | Yes | Yes | Yes | Yes | Yes | Yes | Yes | Yes | Yes | Inclued |
| Lam,et al. | Yes | Yes | Yes | No | Yes | Yes | Yes | Yes | Yes | Yes | Inclued |
| Wang,et al. | Yes | Yes | Yes | Yes | Yes | Yes | No | Yes | Yes | Yes | Inclued |
| Cheah,et al. | Yes | Yes | Yes | Yes | Yes | Yes | No | Yes | Yes | Yes | Inclued |
| de la Fuente, et al. | Yes | Yes | Yes | Yes | Yes | Yes | Yes | Yes | Yes | Yes | Inclued |
| Taoka,et al. | Yes | Yes | Yes | Yes | Yes | Yes | Yes | Yes | Yes | Yes | Inclued |
| Baron,et al. | Yes | Yes | Yes | Yes | Yes | Yes | Yes | Yes | Yes | Yes | Inclued |
| Klimova,et al. | Yes | Yes | Yes | Yes | Yes | Yes | Yes | Yes | Yes | Yes | Inclued |
| Lee,et al. | Yes | Yes | Yes | Yes | Yes | Yes | Yes | Yes | Yes | Yes | Inclued |
| Teckie,et al. | Yes | Yes | Yes | Yes | Yes | Yes | Yes | Yes | Yes | Yes | Inclued |
| Castellino,et al. | Yes | Yes | Yes | Yes | Yes | Yes | Yes | Yes | Yes | Yes | Inclued |
| Gozzi,et al. | Yes | Yes | Yes | Yes | Yes | Yes | Yes | Yes | Yes | Yes | Inclued |
| Kim,et al. | Yes | Yes | Yes | Yes | Yes | Yes | Yes | Yes | Yes | Yes | Inclued |
| Riemens,et al. | Yes | Yes | Yes | Yes | Yes | Yes | Yes | Yes | Yes | Yes | Inclued |

Numbers for questions Q1–Q10 in heading refer to: Q1, were there clear criteria for inclusion in the case series? Q2, was the condition measured in a standard, reliable way for all

participants included in the case series? Q3, were valid methods used for identification of the condition for all participants included in the case series? Q4, did the case series have

consecutive inclusion of participants? Q5, did the case series have complete inclusion of participants? Q6, was there clear reporting of the demographics of the participants in the study?

Q7, was there clear reporting of clinical information of the participants? Q8, were the outcomes or follow up results of cases clearly reported? Q9, was there clear reporting of the presenting

site(s)/clinic(s) demographic information? Q10, was statistical analysis appropriate?
